# Rapid and selective actuation of 3D-printed shape-memory composites via microwave heating

**DOI:** 10.1038/s41598-023-45519-z

**Published:** 2023-10-24

**Authors:** Soo-Chan An, Yeonsoo Lim, Young Chul Jun

**Affiliations:** Department of Materials Science and Engineering, Ulsan National Institute of Science and Technology (UNIST), Ulsan, 44919 Republic of Korea

**Keywords:** Mechanical engineering, Materials science

## Abstract

Three-dimensional (3D) printing allows the fabrication of complex shapes with high resolutions. However, the printed structures typically have fixed shapes and functions. Four-dimensional printing allows the shapes of 3D-printed structures to be transformed in response to external stimuli. Among the external stimuli, light has unique advantages for remote thermal actuation. However, light absorption in opaque structures occurs only near the sample surface; thus, actuation can be slow. Here, we propose and experimentally demonstrate the rapid and selective actuation of 3D-printed shape-memory polymer (SMP) composites using microwave heating. The SMP composite filaments are prepared using different amounts of graphite flakes. Microwave radiation can penetrate the entire printed structures and induce rapid heating. With sufficient graphite contents, the printed SMP composites are heated above their glass transition temperature within a few seconds. This leads to rapid thermal actuation of the 3D-printed SMP structures. Finally, dual-material 3D printing is demonstrated to induce selective microwave heating and control actuation motion. Our experiments and simulations indicate that microwave heating of SMP composites can be an effective method for the rapid and selective actuation of complex structures.

## Introduction

Three-dimensional (3D) printing allows the bottom-up fabrication of complex shapes with high resolutions using various materials, such as metal powders, polymers, ceramics, and composites. A variety of 3D-printing methods have been developed, including fused deposition modeling (FDM), stereolithography, direct ink writing, and selective laser sintering. 3D printing has become popular in various industries and research fields. However, the shapes and functions of most 3D-printed structures are usually fixed once they are printed. Four-dimensional (4D) printing adds active and responsive functions to 3D-printed structures^1–11^. Active responses can be programmed into materials via structural and compositional design. By printing smart materials or multiple materials with different material responses, 4D printing allows the shapes or properties of 3D-printed structures to be transformed in response to external stimuli such as humidity, heat, light, pH, and electric or magnetic fields^12–29^. These active printed structures have various potential applications in actuators, deployable structures, soft robotics, and medical devices^30–35^.

Shape-memory polymers (SMPs) are one of the most widely used smart materials for 4D printing. They consist of solid parts and deformable molecular chains and typically remain rigid at room temperature^36^. They can have a transition from rigid to rubbery states above the glass transition temperature T_g_. Using this property, thermomechanical programming can be performed; a temporary shape can be formed above T_g_ and fixed by cooling it back to room temperature. The original shape can be recovered when the material is heated above T_g_ again because the locked molecular chains become mobile above T_g_. Numerous SMPs are thermoplastic materials that can be employed in conventional FDM. In FDM 3D printing, thermoplastic materials are melted and extruded through a nozzle. They are then solidified and piled layer-by-layer to form 3D objects. This method is widely used in both low-cost and professional 3D printers.

Among the different types of external stimuli, light has unique advantages for remote non-contact actuation^15,16,37–45^. Various properties of light (intensity, polarization, or color) can be rapidly and precisely adjusted to control the responses of printed structures. Additionally, light can be focused onto a small spot, allowing local heating and remote actuation with high resolutions in space and time. Alternatively, sunlight can be utilized to deploy 3D-printed SMPs without an additional energy source^16^. However, 3D-printed materials are often *opaque*; thus, the incident light cannot directly penetrate the printed materials and can only be absorbed near the surface. Then, actuation under light illumination can be slow, taking up to a few minutes^16^. This slow speed can be a limiting factor for actuator applications.

Here, we propose and experimentally demonstrate the rapid actuation of 3D-printed SMP composites using microwave radiation instead of light. SMP composite filaments are prepared using different amounts of carbon materials (graphite flakes) and are 3D-printed using a standard FDM printer. Microwave radiation can penetrate the entire printed structures and induce rapid heating of SMP composites. The printed SMP composites can be rapidly heated above their T_g_ values within a few seconds if graphite contents are sufficient. This leads to rapid actuation of the 3D-printed structures. Finally, dual-material FDM printing is demonstrated to induce selective microwave heating and control actuation motion. Selective heating can be realized in our case using proper multi-material structures with graphite composite and pure PLA parts, where only the graphite composite part is selectively heated by microwave radiation. Hand-shaped structures (rock–paper–scissors) are created by printing the SMP composites and pure SMPs together. Selective actuation of the composite part is demonstrated under microwave radiation. Furthermore, our experiments are supported by numerical simulations. Rapid heating under microwave radiation is modeled using the finite-element method (FEM). In addition, the penetration of the printed composite samples by microwave radiation is analyzed via finite-difference time-domain (FDTD) simulation. Our experiments and simulations indicate that the microwave heating of SMP composite structures can be an effective method for the rapid and selective thermal actuation of complex structures.

## Results and discussion

SMP composite filaments were prepared using different amounts of graphite flakes in a SMP matrix. Figure S1 in Supplementary Information shows images of the fabrication procedure. Poly(lactic acid) (PLA) was used as the SMP matrix. First, the graphite flakes were dissolved in dichloromethane (DCM) and sonicated in an ultrasonic cleaner (KODO Technical Research Co., Ltd., NXP-1002) for 10 min to obtain a uniform dispersion. PLA pellets were dissolved in the same DCM solvent and blended for 90 min (Fig. S1a). After the PLA solution was uniformly mixed with graphite flakes, the solution was solidified in a fume hood (Fig. S1b). The DCM solvent was evaporated, and the solution was completely solidified in 8 h. The solidified composites were chopped into small pieces (Fig. S1c), and the composite filaments were finally obtained using a filament extruder (Noztek) (Fig. S1d). It was important to maintain a uniform filament size during extrusion. A composite filament with a uniform diameter (~ 1.75 mm) was obtained by monitoring the filament height and keeping the extrusion speed constant. The red dashed box in Fig. S1d shows a height sensor for the filament.

**Figure 1:**
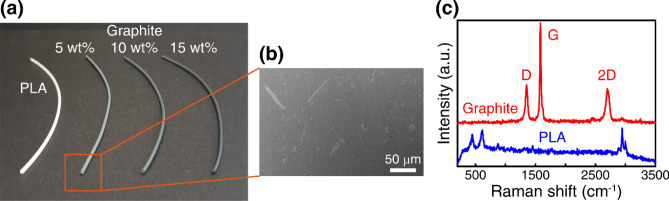
Fabrication and characterization of shape-memory polymer (SMP) composite filaments. (**a**) Picture of the composite filaments. (**b**) SEM image of the 5 wt% filament. (**c**) Th Raman spectra measured from the cross section of the composite filament show two distinct regions, which correspond to the graphite flakes and PLA. In general, the combination of these two spectra appears in the composite filaments.

Figure 1a shows the picture of the prepared composite filaments with different graphite contents (5, 10, and 15 wt% graphite flakes in PLA). The filament becomes darker with an increase in the graphite content. A pristine PLA filament (i.e. without graphite flakes) is shown for comparison in Fig. 1a (white). Figure 1b presents a cross-sectional scanning electron microscopy (SEM) image of the composite filament with 5 wt% graphite. As shown, the graphite microflakes are dispersed in the PLA matrix. The cross section of the composite filament was also investigated using Raman scattering measurements. The Raman scattering spectrum was measured using a 532-nm laser in a commercial confocal Raman setup (WITec alpha300R). When a focused laser spot was moved across the cross section of the composite filament, two different types of Raman spectra could be observed (Fig. 1c), which correspond to the graphite flakes and PLA^46–48^. In general, the combination of these two spectra appeared in the composite filaments, confirming the formation of the composite in the filament.

**Figure 2:**
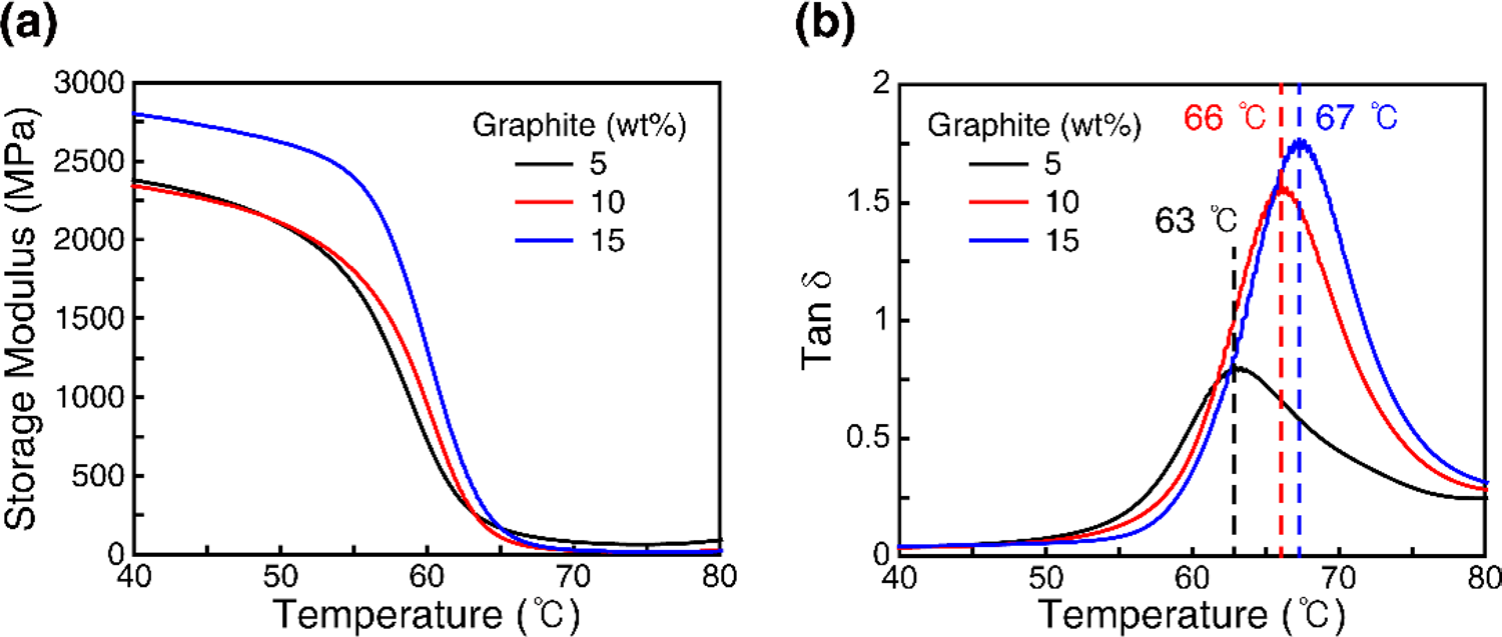
Characterization of SMP composite filaments. (**a**) Storage modulus of the composites. (**b**) Loss factor (tanδ). The peak of the loss factor appears at a glass transition temperature.

The material response of the composite filaments was characterized using dynamic mechanical analysis (DMA). A composite SMP plate with dimensions of 10 mm × 3 mm × 1 mm was printed using an FDM 3D printer (MakerBot Replicator 2X). The storage modulus was measured using DMA as a function of temperature under 1-Hz oscillation. Figure 2 shows the measured data for the 5, 10, and 15 wt% samples. The storage modulus decreased sharply to low values at approximately 60–70 °C. This indicates that the SMP composite samples soften above this temperature range and can recover their original shapes. For the considered range of graphite contents, the storage modulus increased with the graphite content. This is attributed to the fact that the dispersed graphite flakes in the SMP matrix functioned as stiff fillers, and the chain mobility of the SMP was more limited at higher graphite contents^49^. To determine the T_g_ values of the composite samples, the loss factor (tanδ) was also obtained (Fig. 2b). The peak of the loss factor indicates T_g_. We find that T_g_ appears approximately 63–67 °C for the three composite filaments.

**Figure 3:**
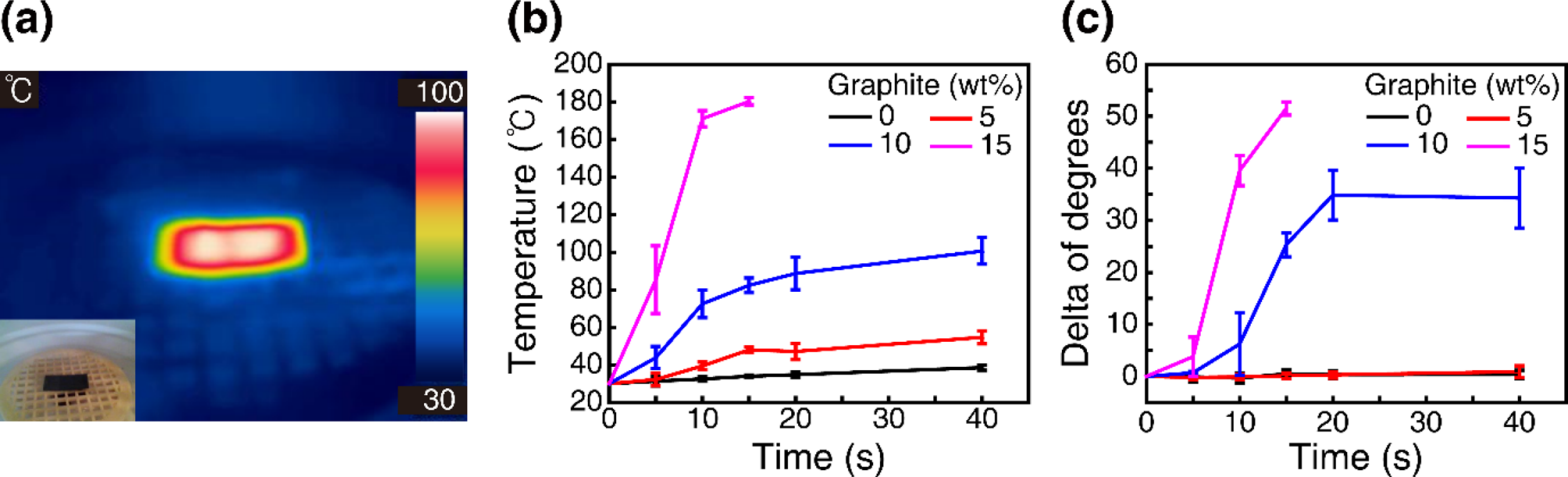
Microwave heating and shape recovery of SMP composite plates. (**a**) Infrared thermal image of the 15 wt% composite sample. Inset: Optical camera image of the sample. (**b**) Sample temperature vs microwave heating time. (**c**) Shape recovery (change in a bent angle) vs microwave heating time. The samples were initially bent at 52°. The error bars were determined from the repetition of measurements.

In our experiment, we used a conventional microwave oven with a frequency of 2.45 GHz and power of 360 W to investigate the thermal responses and shape-memory effects of the SMP composites^50^. For the microwave heating experiments, composite SMP plates with 5, 10, and 15 wt% graphite flakes were printed again with dimensions of 40 mm × 20 mm × 1 mm. The printed samples were placed on a meshed holder inside the microwave oven (see the inset of Fig. 3a). The typical rotating glass plate in the microwave oven was replaced by the meshed holder, which kept the sample fixed without rotation. The meshed holder was 3D-printed using acrylonitrile butadiene styrene (ABS), which has a higher T_g_ than PLA and remained stable during the experiment. The samples were heated for up to 40 s in 5 s increments, and the sample temperatures were monitored using a thermal imaging camera (FLIR-C2, FLIR). Figure 3a presents an example thermal infrared image of the 15 wt% sample. The scale on the right side indicates the local temperature. As shown, the SMP composite was heated under microwave radiation, whereas the ABS mesh holder remained cool. Figure 3b shows how the temperature of the SMP composites changes over microwave time. Three SMP composite samples (5, 10, and 15 wt%) and a pure PLA sample (0 wt%) were compared. The measured data indicated that a composite plate with a higher graphite content was heated faster. The 15 wt% sample was heated above T_g_ (67 °C) within a few seconds. The temperature of the 15 wt% sample increased so rapidly that the sample started to melt beyond 15 s. The 10 wt% sample was heated above T_g_ within 10 s, and its temperature increased steadily during the measurements. However, the 5 wt% and pure PLA samples did not reach T_g_ during our microwave time.

To characterize the shape-memory effect of the SMP composites, the printed plates were bent at 52° in hot water (80 °C) and then fixed in cold water. Subsequently, the shape recovery (i.e. bending-angle change) of the bent samples was monitored. Figure 3c shows the change in the bending angle over microwave time. The 15 and 10 wt% composite samples exhibited rapid shape recovery as they were quickly heated above T_g_, whereas the 5 wt% and pure PLA samples did not exhibit shape recovery, because they were not heated above T_g_. The measurements shown in Fig. 3 verify that rapid microwave heating and shape recovery can be achieved in SMP composites with sufficient graphite contents. In addition, the variation of the graphite content in SMP composites leads to a sharp contrast in the heating and shape recovery of the SMP. As will be demonstrated later, this large contrast can be utilized in multi-material 4D printing to realize controlled shape changes.

**Figure 4:**
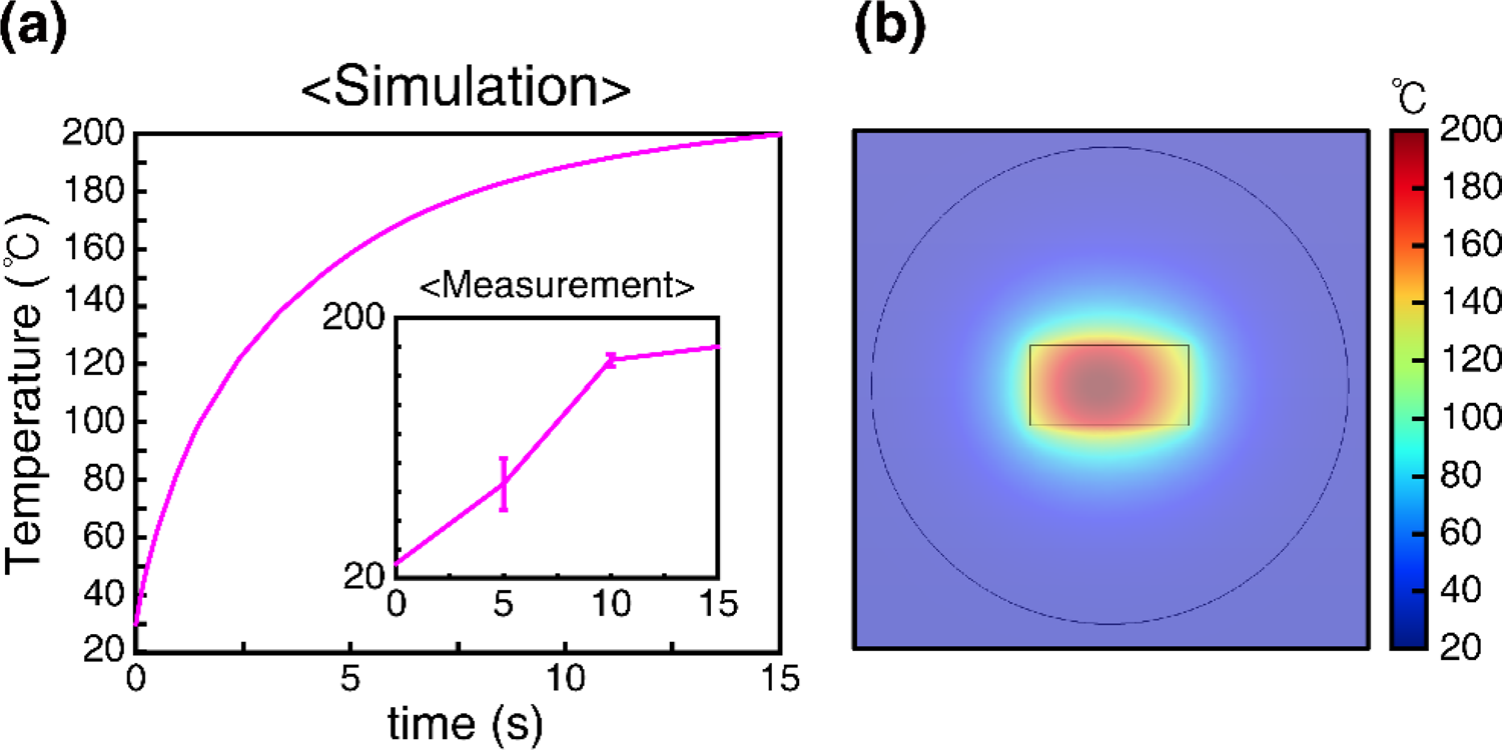
Simulation of microwave heating (15 wt% composite plate). (**a**) Sample temperature vs microwave heating time. Inset: Experimental data from Fig. 3b. (**b**) Simulated temperature distribution around the sample.

To confirm the results of our microwave heating experiments, we conducted COMSOL finite-element analyses and simulated the temperature change of the 15 wt% sample in the microwave oven. The complex refractive index of the 15 wt% sample was determined at the Korea Research Institute of Standards and Science (KRISS) using the split post-dielectric resonator method (*n* = 3.2098 + 0.1245i at 3 GHz). In this method, the complex refractive index is determined from the resonance frequency and sample dimensions. The measured optical constant indicates that the SMP composite is a lossy *dielectric* material (not metallic) in the considered microwave range. In the FEM simulation, the microwave oven was modeled as a cavity with dimensions of 357 mm × 356 mm × 240 mm and a microwave source of 360 W at 2.45 GHz. The composite plate was placed on top of an ABS mesh holder (radius = 60 mm, height = 3.2 mm), and a heat transfer to air was introduced. The ABS mesh was modeled as a plate with an average refractive index of 1.23, which was determined by considering the ratio of the ABS part to the empty region in the mesh. Figure 4a shows the simulated temperatures of the composite sample. The simulated temperature of the sample rapidly increased within a few seconds, as observed in the experiment. For comparison, the experimentally measured temperature of the 15 wt% sample is shown in the inset. In the simulation, the temperature initially increased rapidly and then became saturated at a higher temperature. Figure 4b shows the overall temperature distribution around the sample after 15 s. In the simulation, the whole composite plate was heated, and the center of the sample reached a high temperature (> 180 °C), in agreement with our experiment (Fig. 3b).

**Figure 5:**
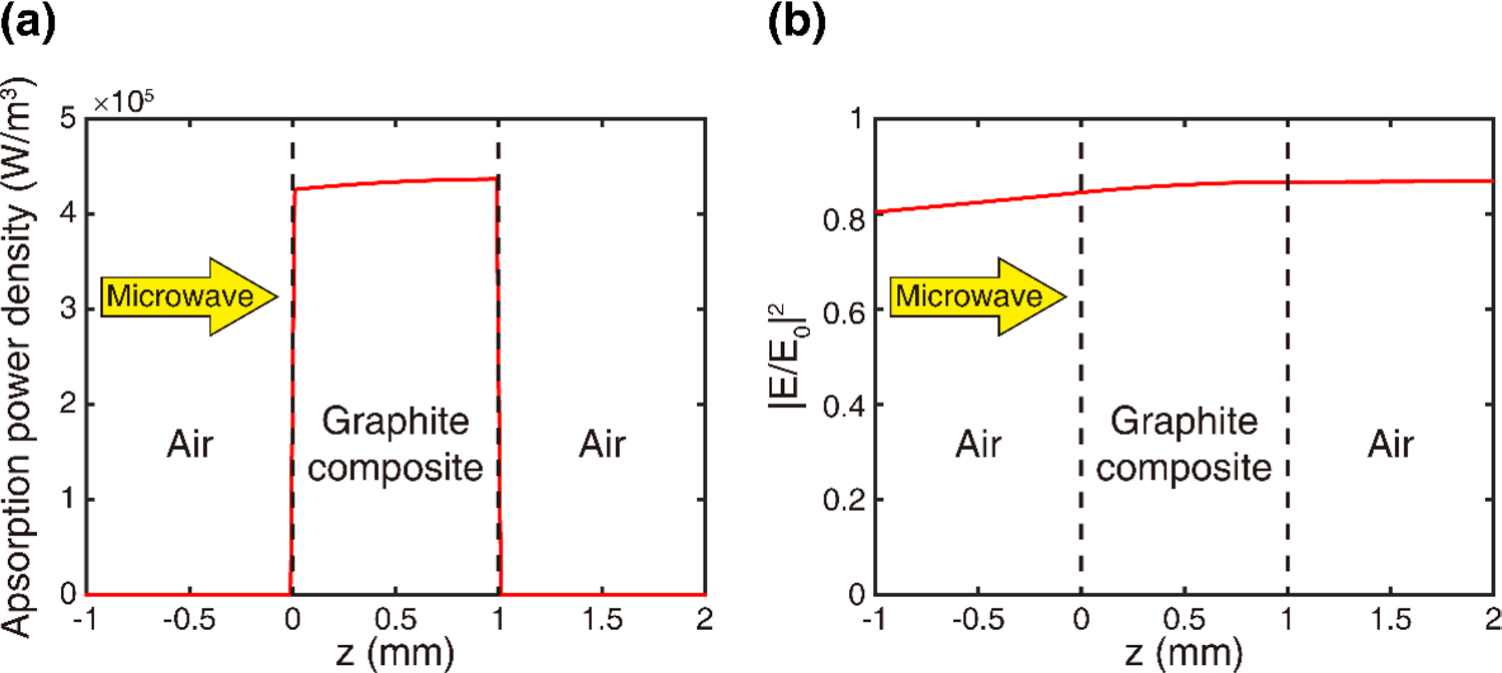
Simulation of microwave penetration. (**a**) Absorption power density and (**b**) Electric field magnitude through a 1-mm-thick composite plate.

To verify that microwave radiation can penetrate the entire composite plate, we conducted another simulation. The absorption power density and the magnitude of the electric field passing through the composite plate were calculated using the FDTD method. The absorption power density of the incident microwave radiation was calculated using the measured refractive index of the 15 wt% sample. The absorption power per unit volume can be calculated from the divergence of the Poynting vector $\overrightarrow{S}$ as follows^51^,

1 $${P}_{abs}=\left|\frac{1}{2}Re\left[\overrightarrow{\nabla }\cdot \overrightarrow{S}\right]\right|=\frac{1}{2}\omega Im\left[{\varepsilon }_{0}\varepsilon (\omega )\right]{\left|E(\omega )\right|}^{2},$$

where *ω* = 2π*f* represents the angular frequency (*f* = 2.45 GHz), *ε*_0_ is the vacuum permittivity, *ε* is the complex dielectric constant, and *E* represents the electric-field magnitude. In the calculation, a 1-mm-thick composite plate was assumed. A plane wave was incident from the left side, as shown in Fig. 5.

Figure 5a indicates that the absorption of microwave radiation occurs over the entire plate (not just near the surface) because the microwave radiation penetrates deeper into the composite sample. The absorption density remains similar throughout the thickness of the sample. The total absorption of the 1-mm-thick composite plate was determined to be 4.3%. Figure 5b shows the electric-field magnitude in the composite plate, which indicated that the microwaves penetrate the entire plate. This simulation implies that microwave radiation can be applied to more complex SMP composite structures for heating the entire printed structure and inducing rapid actuation. In contrast, light absorption for opaque materials typically occurs only near the sample surface. This can lead to slow actuation under light illumination.

**Figure 6:**
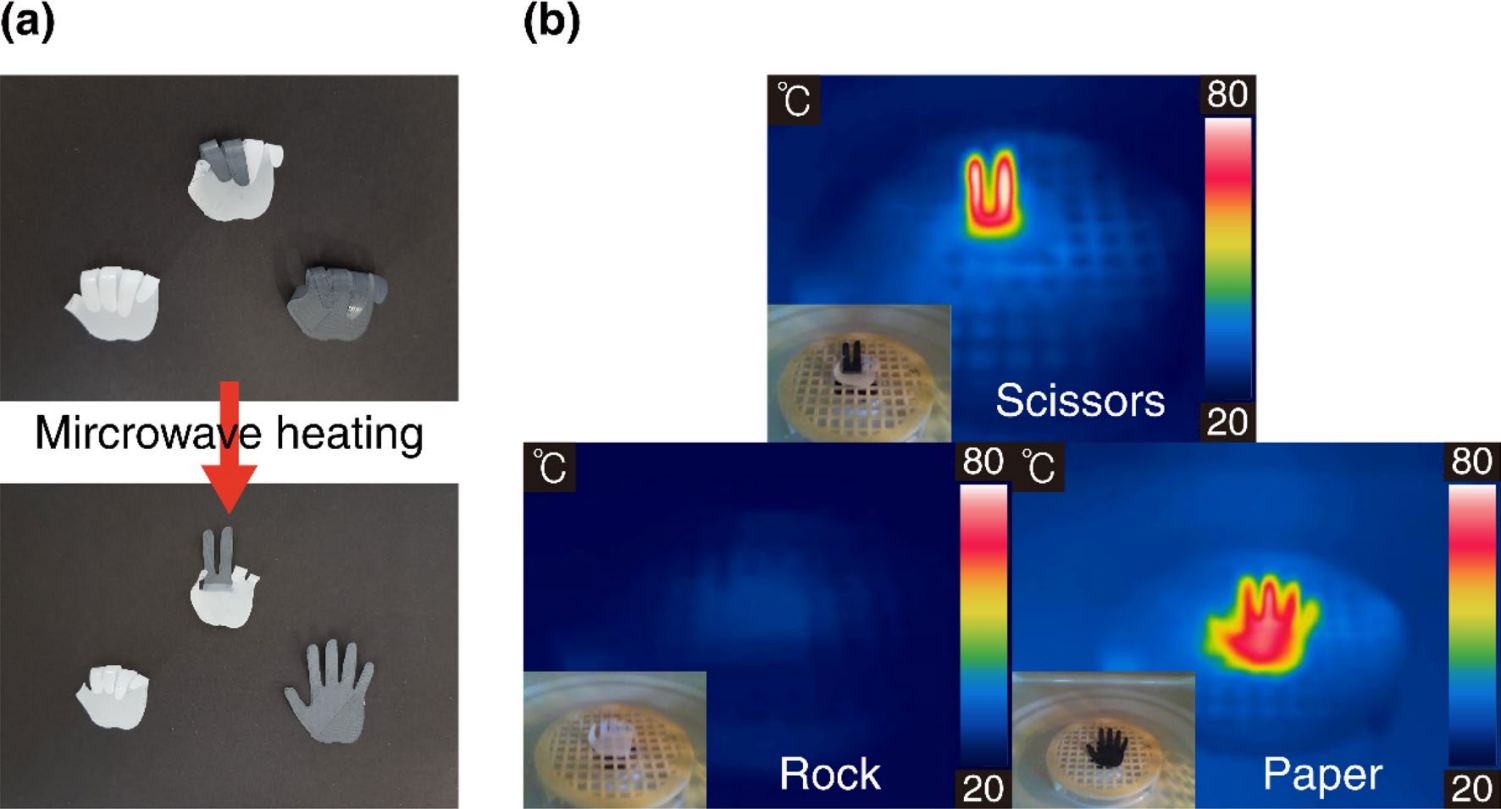
Dual-material 3D printing for selective microwave heating and control of actuation motion. (**a**) Picture of three hand-shaped structures (rock–paper–scissors). (**b**) Infrared thermal images of the three samples.

Finally, dual-material FDM printing (MakerBot Replicator 2X) was employed to demonstrate selective microwave heating and control of actuation motion. Three hand shapes were 3D-printed using two different filaments (10 wt% composite and pure PLA) (Fig. 6a). The 10 wt% composite SMP is dark gray in color, whereas the pure PLA appears white. Because only the 10 wt% composite is heated in a reasonable time under microwave radiation, the three hand-shaped structures can be transformed differently under microwave heating. Therefore, dual-material (or multi-material) 4D printing can be used to induce selective microwave heating in complex 3D objects, which can lead to controlled actuation motions.

After the thermomechanical programming, all three structures were first bent, as shown in Fig. 6a. After 20 s of heating in the microwave oven, the three structures were transformed into different shapes: rock (PLA only), paper (10 wt% composite on the entire hand), and scissors (10 wt% composite on two fingers only). Figure 6b shows thermal infrared images and corresponding optical camera images of the three samples. Only the parts made of the graphite flake composite were heated; thus, the three hand-shaped samples were transformed into different shapes. Thus, we demonstrated rapid and selective heating of 4D-printed structures via microwave radiation. The microwave time was relatively short (20 s), and it can be further shortened by increasing the graphite content. We expect that by using several different compositions and appropriate heating times, more complex sequential actuation can be achieved. For simplicity, we conducted experiments using a microwave oven. However, in principle, well-collimated electromagnetic (microwave) radiation can be directed to a target position through freespace as light can be directed over a long distance. In that case, remote actuation can be realized using microwave radiation. The detailed comparisons of microwave heating with conventional hot-water-based heating^52^ are also provided in Supplementary Information.

## Conclusion

4D printing can enable shape changes via structural and compositional design. Light has advantages for remote thermal actuation. However, for typical opaque structures, light absorption occurs only near the surface. The resulting slow actuation speed can be a limiting factor in actuator applications. In this study, we experimentally demonstrated the rapid actuation of 3D-printed SMP composites using microwave radiation. SMP composite filaments were prepared by employing different amounts of graphite flakes and were 3D-printed using a standard FDM printer. Graphite fillers efficiently absorb microwave radiation and generate large amounts of thermal energy in a short time. In particular, microwave radiation can penetrate the entire printed structure and induce rapid heating of SMP composites. The printed composite structures in our experiments were rapidly heated above their glass transition temperature within a few seconds when the graphite content was sufficient, resulting in rapid actuation. Finally, dual-material FDM printing was used to demonstrate the selective microwave heating and control of actuation motion. The results suggest that more complex actuation motions can be achieved by employing several different compositions and proper heating times. Our experiments and simulations indicate that the microwave heating of SMP composite structures can be an effective method for the rapid and selective actuation of complex structures.

### Supplementary Information

Supplementary Information.

## Data Availability

The raw data and the processed data required to reproduce the findings in the current study are available from the corresponding author on reasonable request.

## References

[1] Tibbits, S. Te emergence of 4D printing. *TED Conferences* (2013).

[2] Kuang X (2019). Advances in 4D printing: Materials and applications. Adv. Func. Mater..

[3] Rastogi P, Kandasubramanian B (2019). Breakthrough in the printing tactics for stimuli-responsive materials: 4D printing. Chem. Eng. J..

[4] Suriano R, Bernasconi R, Magagnin L, Levi M (2019). 4D printing of smart stimuli-responsive polymers. J. Electrochem. Soc..

[5] Mitchell A, Lafont U, Hołyńska M, Semprimoschnig C (2018). Additive manufacturing—A review of 4d printing and future applications. Addit. Manuf..

[6] Wu J-J, Huang L-M, Zhao Q, Xie T (2018). 4D printing: History and recent progress. Chin. J. Polym. Sci..

[7] Lee AY, An J, Chua CK (2017). Two-Way 4D printing: A review on the reversibility of 3d-printed shape memory materials. Engineering.

[8] Lee J, Kim H-C, Choi J-W, Lee IH (2017). A review on 3D printed smart devices for 4D printing. Int. J. Precis. Eng. Manuf. Green Technol..

[9] Momeni F, Mehdi Hassani S, Liu X, Ni J (2017). A review of 4D printing. Mater. Des..

[10] Shin D-G, Kim T-H, Kim D-E (2017). Review of 4D printing materials and their properties. Int. J. Precis. Eng. Manuf. Green Technol..

[11] Choi J, Kwon OC, Jo W, Lee HJ, Moon M-W (2015). 4D printing technology: A review. 3D Print. Addit. Manuf..

[12] Behl M, Razzaq MY, Lendlein A (2010). Multifunctional shape-memory polymers. Adv. Mater..

[13] Johnson JA, Turro NJ, Koberstein JT, Mark JE (2010). Some hydrogels having novel molecular structures. Prog. Polym. Sci..

[14] Kuksenok O, Balazs AC (2016). Stimuli-responsive behavior of composites integrating thermo-responsive gels with photo-responsive fibers. Mater. Horiz..

[15] Lendlein A, Jiang H, Jünger O, Langer R (2005). Light-induced shape-memory polymers. Nature.

[16] Yang H (2017). 3D printed photoresponsive devices based on shape memory composites. Adv. Mater..

[17] Zhu C, Bettinger CJ (2014). Light-induced remodeling of physically crosslinked hydrogels using near-IR wavelengths. J. Mater. Chem. B.

[18] Zhu C, Bettinger CJ (2015). Photoreconfigurable physically cross-linked triblock copolymer hydrogels: Photodisintegration kinetics and structure–property relationships. Macromolecules.

[19] Ding Z (2017). Direct 4D printing via active composite materials. Sci. Adv..

[20] Felton SM (2013). Self-folding with shape memory composites. Soft Matter.

[21] Ge Q, Dunn CK, Qi HJ, Dunn ML (2014). Active origami by 4D printing. Smart Mater. Struct..

[22] Kotikian A, Truby RL, Boley JW, White TJ, Lewis JA (2018). 3D printing of liquid crystal elastomeric actuators with spatially programed nematic order. Adv. Mater..

[23] Stroganov V (2014). Biodegradable self-folding polymer films with controlled thermo-triggered folding. Adv. Funct. Mater..

[24] Sydney Gladman A, Matsumoto EA, Nuzzo RG, Mahadevan L, Lewis JA (2016). Biomimetic 4D printing. Nat. Mater..

[25] Raviv D (2014). Active printed materials for complex self-evolving deformations. Sci. Rep..

[26] Cho JW, Kim JW, Jung YC, Goo NS (2005). Electroactive shape-memory polyurethane composites incorporating carbon nanotubes. Macromol. Rapid Commun..

[27] Leng J, Lv H, Liu Y, Du S (2007). Electroactivate shape-memory polymer filled with nanocarbon particles and short carbon fibers. Appl. Phys. Lett..

[28] Lu H, Liu Y, Gou J, Leng J, Du S (2010). Electrical properties and shape-memory behavior of self-assembled carbon nanofiber nanopaper incorporated with shape-memory polymer. Smart Mater. Struct..

[29] Nadgorny M, Xiao Z, Chen C, Connal LA (2016). Three-dimensional printing of pH-responsive and functional polymers on an affordable desktop printer. ACS Appl. Mater. Interfaces.

[30] Wehner M (2016). An integrated design and fabrication strategy for entirely soft, autonomous robots. Nature.

[31] Zarek M (2016). 3D printing of shape memory polymers for flexible electronic devices. Adv. Mater..

[32] Maitland DJ, Metzger MF, Schumann D, Lee A, Wilson TS (2002). Photothermal properties of shape memory polymer micro-actuators for treating stroke. Lasers Surg. Med..

[33] Kuang X (2018). 3D printing of highly stretchable, shape-memory, and self-healing elastomer toward novel 4D printing. ACS Appl. Mater. Interfaces.

[34] Lendlein A, Langer R (2002). Biodegradable, elastic shape-memory polymers for potential biomedical applications. Science.

[35] Malachowski K (2014). Stimuli-responsive theragrippers for chemomechanical controlled release. Angew. Chem. Int. Ed..

[36] Behl M, Lendlein A (2007). Shape-memory polymers. Mater. Today.

[37] Lee Y, Lee H, Hwang T, Lee J-G, Cho M (2015). Sequential folding using light-activated polystyrene sheet. Sci. Rep..

[38] Ahn S-K, Ware TH, Lee KM, Tondiglia VP, White TJ (2016). Photoinduced topographical feature development in blueprinted azobenzene-functionalized liquid crystalline elastomers. Adv. Funct. Mater..

[39] Donovan BR, Matavulj VM, Ahn S-K, Guin T, White TJ (2019). All-optical control of shape. Adv. Mater..

[40] Jeong HY, Woo BH, Kim N, Jun YC (2020). Multicolor 4D printing of shape-memory polymers for light-induced selective heating and remote actuation. Sci. Rep..

[41] Herath M, Epaarachchi J, Islam M, Fang L, Leng J (2020). Light activated shape memory polymers and composites: A review. Eur. Polym. J..

[42] Shimoga G, Choi D-S, Kim S-Y (2021). Bio-inspired soft robotics: Tunable photo-actuation behavior of azo chromophore containing liquid crystalline elastomers. Appl. Sci..

[43] Jeon J (2021). Continuous and programmable photomechanical jumping of polymer monoliths. Mater. Today.

[44] Lu X (2021). 4D-printing of photoswitchable actuators. Angew. Chem. Int. Ed..

[45] Jeong HY, An S-C, Jun YC (2022). Light activation of 3D-printed structures: from millimeter to sub-micrometer scale. Nanophotonics.

[46] Ammar MR (2015). Characterizing various types of defects in nuclear graphite using Raman scattering: Heat treatment, ion irradiation and polishing. Carbon.

[47] Greco C (2019). Role of the carbon defects in the catalytic oxygen reduction by graphite nanoparticles: A spectromagnetic, electrochemical and computational integrated approach. Phys. Chem. Chem. Phys..

[48] Cuiffo MA (2017). Impact of the fused deposition modeling (FDM) printing process on polylactic acid (PLA) chemistry and structure. Appl. Sci..

[49] Guo R, Ren Z, Bi H, Xu M, Cai L (2019). Electrical and thermal conductivity of polylactic acid (PLA)-based biocomposites by incorporation of nano-graphite fabricated with fused deposition modeling. Polymers.

[50] Koh TY, Sutradhar A (2022). Untethered selectively actuated microwave 4D printing through ferromagnetic PLA. Addit. Manuf..

[51] Jackson JD (1999). Classical Electrodynamics.

[52] Narumi K (2023). Inkjet 4D print: Self-folding tessellated origami objects by inkjet UV printing. ACM Trans. Graph..

