# Supplementary material for: Rapid and selective actuation of 3D-printed shape-memory composites via microwave heating
Source: Sci Rep. 2023 Oct 24;13:18179. doi: 10.1038/s41598-023-45519-z (PMC10598202; doi:10.1038/s41598-023-45519-z)
Supplement: Supplementary file 1 — Supplementary Information. [file 41598_2023_45519_MOESM1_ESM.docx]

Supplementary Information for

**Rapid and selective actuation of 3D-printed shape-memory composites via microwave heating**

Soo-Chan An^1^, Yeonsoo Lim^1^, and Young Chul Jun^1^

^1^Department of Materials Science and Engineering, Ulsan National Institute of Science and Technology (UNIST), Ulsan 44919, Republic of Korea


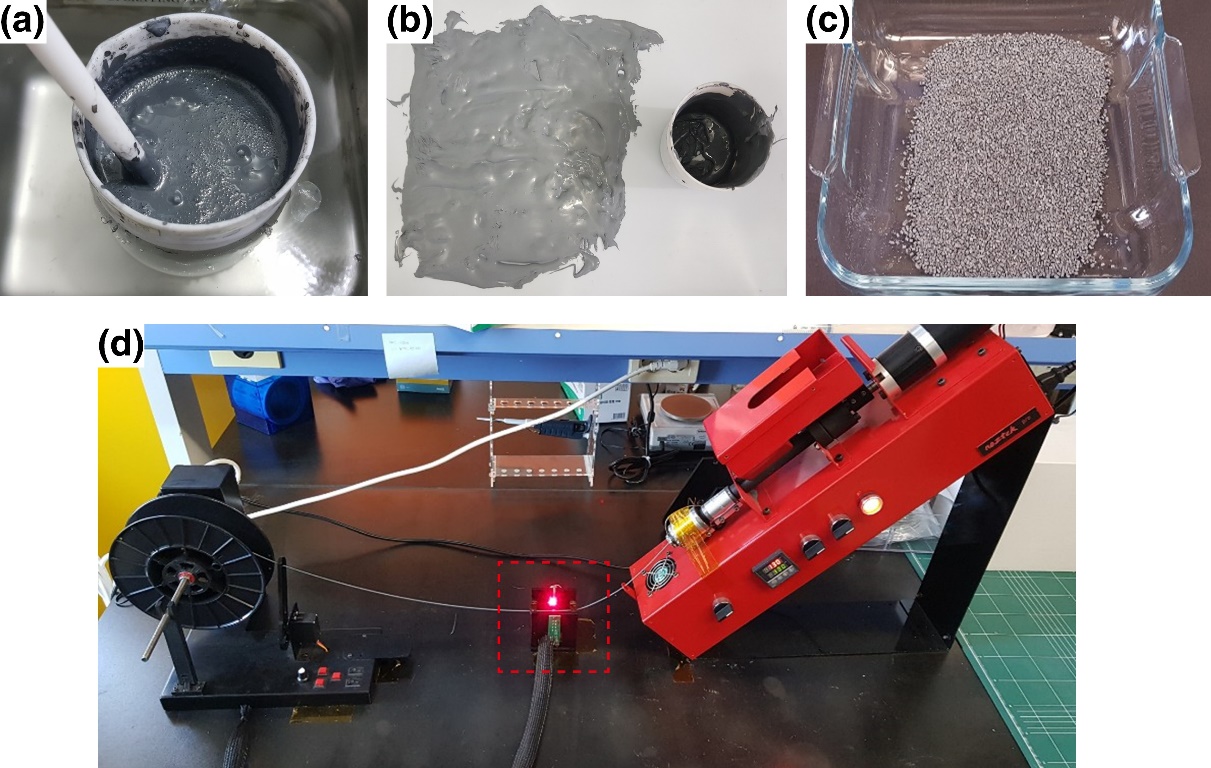


**Figure S1. Fabrication procedure of shape-memory polymer (SMP) composite filaments.** (a) Poly(lactic acid) (PLA) pellets and graphite flakes are dissolved in dichloromethane (DCM). (b) The mixture solution is solidified in a fume hood. (c) The solidified composites are chopped into small pieces. (d) Composite filaments are obtained using a filament extruder. The red dashed box shows a height sensor for the filament.

**Comparison of microwave heating with conventional hot-water-based heating**

Here, we provide the detailed comparisons of microwave heating with conventional hot-water-based heating.

**1) Selective heating**

When a hand-shaped, dual-material structure is immersed in hot water, both graphite composite (two figures) and pure PLA parts are uniformly heated and actuated (Fig. S2). This clearly demonstrates the idea of selective heating in our approach based on microwave heating.

When a dual-material structure is prepared using PLA and ABS and heated in hot water, the entire structure is still *uniformly* heated, but only the PLA part can be selectively actuated because of the T_g_ difference. In the case of microwave heating, although we did not focus the microwave radiation onto a specific position, proper *multi-material* structures can be selectively heated and actuated. Figure 6b in the main text shows such a case. For the hand-shaped structure made of the graphite composite (two figures) and pure PLA, only the two figures are “selectively” heated and actuated (red-white color in thermal infrared image) while the other part remains cool (blue color). Indeed, a temperature contrast between the graphite composite and pure PLA parts is very large.


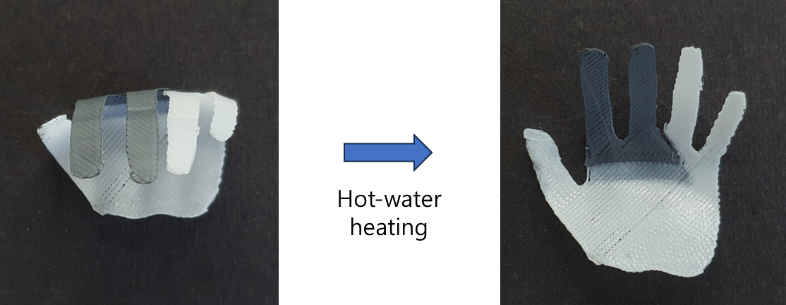


**Figure S2. Hot-water-based thermal actuation at 67 °C**

**2) Speed**

In Fig. S2, a hand-shaped structure is actuated in 5 ~ 6 seconds at 67 °C (T_g_) using hot-water-based heating. For very thin structures and at higher temperatures, the speed of actuation can be even faster. In a practical sense, we think both microwave and hot-water heating can enable rapid actuation.

In some cases, we may also want to precisely control the speed of actuation at our target value. We find that microwave heating of composite structures has continually variable control parameters (such as microwave power, microwave time, graphite weight percent, etc). These multiple tuning parameters can be used together to enable precise control of actuation speed and motion.

**3) Efficiency**

A microwave source of 360 W consumes 360 J of energy per second. In the case of the 15 wt% composite, we can heat up the composite structure above T_g_ within 5 seconds. Therefore, we need less than 1800 J for microwave heating. (In fact, only part of this microwave energy was delivered to the hand-shaped structure inside the microwave oven)

On the other hand, the specific heat capacity c_p_ of liquid water at constant pressure is about 4.187 J/(g·°C). The size of the hand-shaped structure is about 5 cm × 5.5 cm, and its height is 1 mm. Thus, its volume is 2.75 cm^3^. Then, to increase the temperature of water from 20 °C to 67 °C (T_g_), we need about 541 J of energy. However, practically, to actuate an initially bent structure, we surely need a much larger volume of hot water in a container. If we immerse the bent hand-shaped structure in hot water of the volume of 5 cm × 5.5 cm × 1 cm, the required energy becomes 5410 J. Therefore, the required energy varies depending on the volume of hot water in the container. In a practical sense, the required energies for microwave and hot-water heating are comparable.

**4) Practical benefit**

For simplicity, we conducted experiments using a microwave oven. However, in principle, well-collimated electromagnetic (microwave) radiation can be directed to a target position through freespace as light can be directed over a long distance. In that case, the range of remote actuation can be much larger compared to other heating methods (such as heat gun or steam). In the case of the steam, we may also need a special guiding channel to direct the steam over a long distance.

In addition to potential remote actuation, microwave heating of graphite composites can allow ‘continually variable’ control parameters (such as graphite weight percent, microwave power, microwave time, etc). This controllability is of practical benefits.
